# The Hierarchy of Communication Needs: A Novel Communication Strategy for High Mistrust Settings Developed in a Brazilian COVID-ICU

**DOI:** 10.1089/pmr.2023.0070

**Published:** 2024-02-09

**Authors:** Daniel Neves Forte, Mark Stoltenberg, Sabrina Correa da Costa Ribeiro, Ingrid Maria (Mia) Olsen de Almeida, Vicki Jackson, Bethany-Rose Daubman

**Affiliations:** ^1^ Intensive Care Unit, Medical Emergency Department, University of São Paulo Medical School, São Paulo, Brazil.; ^2^ Teaching and Research Institute, Sírio Libanês Hospital, São Paulo, Brazil.; ^3^ Division of Palliative Care and Geriatric Medicine, Massachusetts General Hospital, Boston, Massachusetts, USA.; ^4^ Harvard Medical School, Boston, Massachusetts, USA.

**Keywords:** Brazil, communication, education, global health, intensive care, Latin America, low- and middle-income country, palliative care, shared-decision making

## Abstract

**Context:**

The COVID-19 pandemic presented unique challenges for health care systems. Overcrowded units, extreme illness severity, uncertain prognoses, and mistrust in providers resulted in a “pressure cooker” where traditional communication strategies were often insufficient.

**Objectives:**

Building on well-studied traditional communication interventions, neurobiology principles were used to create a novel communication strategy designed in the COVID-ICU to respond to the unique communication needs of patients within the context of a high mistrust setting.

**Methods:**

The hierarchy of communication needs recognizes three specific levels of communication that are essential within high-emotion and low-trust settings. The first level is to establish trust. The second level is to resonate with patients' emotions, helping to reduce arousal and improve empathy. The third level includes the more traditional content of disclosing prognostic information and shared decision-making. When facing communication challenges, clinicians are taught to move back a level and reattune to emotions and/or reestablish trust.

**Discussion:**

The COVID pandemic revealed the shortcomings of a primarily cognitive communication style. The hierarchy of communication needs emphasizes trust building, and emotional resonance as prerequisites of effective cognitive discussions, resulting in more effective clinician–patient communication that more fully incorporates cultural humility and better meets the needs of diverse patient populations. Additional research is needed to further develop this strategy and evaluate its impact on patient experience and outcomes.

## Background: A Novel Communication Strategy Formed in a Brazilian ICU at the Peak of the COVID Pandemic

The unique context of Brazil at the peak of the COVID pandemic revealed the shortcomings of a purely cognitive communication style rooted in the western biomedical model. Due to the severity of illness, substantial symptom burden, significant clinical uncertainty, and feelings of fear and insecurity experienced by patients, families, and clinicians, a fundamental breakdown in trust was occurring. This lack of trust resulted in strong emotions on all sides, making communication with patients and families uniquely challenging.

Though it was exacerbated by the unique factors of a COVID-19 ICU at the peak of the pandemic, the challenge of mistrust between patients and clinicians certainly predates the pandemic. This is especially true for members of marginalized groups, where distrust of medical institutions can be deeply seeded due to painful historical and ongoing trauma and systemic injustice. Compounding the long-standing presence of systemic mistrust in Brazil, the unique pressure cooker environment of the COVID ICU at the height of the pandemic forced clinical teams to develop a novel communication strategy to establish trust and attune to patient emotions before moving toward shared decision-making. Though it builds on other well-studied communication skills, this hierarchy of communication needs describe a novel cognitive map to assist clinicians facing high-pressure and low-trust settings like the Brazilian ICU during the COVID pandemic. Since its initial creation, this communication framework has been further developed into a formal teaching structure together with collaborators from the University of São Paulo Medical School, Sirio-Libanês Hospital, and the Massachusetts General Hospital Global Palliative Care (PC) Program. This article seeks to describe this novel communication frame first developed and implemented in Brazil.

## The Hierarchy of Communication Needs: A Biological Approach to Communication Within High-Mistrust Settings

**FIG. 1.**
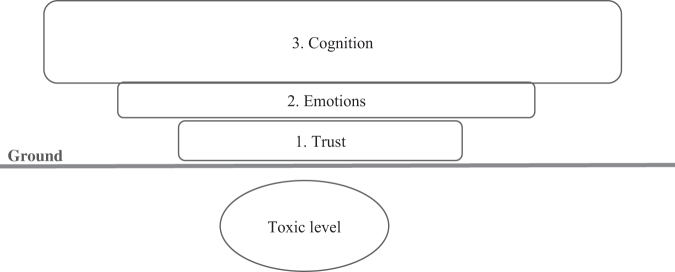
Hierarchy of communication needs: three levels of complexity in interpersonal communication.

Due to the frequent presence of strong emotions and mistrust between patients and clinicians within the context of the COVID-19 ICU, more traditional communication strategies focusing on the rational sharing of goals and values were ineffective. Clinicians recognized that they needed to establish trust and manage strong emotions before attempting the more cognitive task of shared decision-making. Additionally, any new model needed to be simple and teachable for busy and stressed ICU clinicians to integrate it meaningfully. These tasks are best described as a hierarchy of communication needs, starting with the foundational need for trust, followed by the need to connect by resonating with emotions. Only once these two levels are addressed can the cognitive activities of shared decision-making be done effectively (Fig. 1). Describing these components as sequential layers enables clinicians to move between levels, typically starting from the basic and moving toward the more complex. For example, when facing communication difficulties within Level 3, clinicians must return to Level 2 with emotional resonance. When necessary, they may need to “retreat” even further back to reestablish trust before moving forward again. Though each level of the hierarchy depends on the levels below it, the size of the levels is drawn inversely to emphasize that, from a neurobiological perspective, the degree of complexity of each level increases as you move higher. It also illustrates for learners that trust and resonating with emotions serve as an essential foundation for effective communication, although clinicians often pay more attention to the more complex cognitive discussions that occur at Level 3.

This approach also follows a clear biological basis rooted in the evolution of our nervous system. The cognitive tasks that are often the exclusive focus of traditional communication strategies require the use of the frontal cortex. However, it is well documented that when faced with stress, strong emotions arising from the amygdala and limbic system can be extremely potent drivers of human behavior. These reactions are often fast and effortless—but are also more likely to be inaccurate when compared with slower, more intentional cognitive responses.^1^ However, emotional valence can also provide helpful cues for appropriate behavior, resulting in better adaptation to changing environments and improved social behavior.^2–5^ Following this neurobiological frame, communication with patients facing serious illness can be improved by recognizing and responding to strong emotions and potential mistrust before moving on to more cognitive tasks.

### Level 1: Establishing trust

Trust is the basis of cooperative behavior to achieve consensus. It is well documented that even the most basic interactions among apes and other social animals are dependent on trust.^2,3^ Without first establishing trust, patients will not share their fears and may remain skeptical of recommendations made by clinicians.^1^ Though trust is a complex and dynamic concept, within the high-intensity and time-sensitive ICU environment, the root cause of patient mistrust was often encountered as a fear of being deceived or of receiving suboptimal care. In response to these fears, clinicians can earn trust from their patients by reviewing their commitment to tell the truth even when it might be difficult and their commitment to do their best to help.^6^ Example language for how clinicians could describe these core values to patients to help establish trust is found in Table 1. These statements can then be followed by a confirmatory question, such as “Does this sound ok to you?” This provides an opportunity for further dialogue and helps enhance trust further, inviting the patient into the conversation. These example phrases can be adapted by individual clinicians, as emotional resonance is stronger when clinicians use their own words.^7^ These statements may not always be necessary under more routine and lower-intensity circumstances. However, when mistrust is suspected, these simple actions can significantly improve communication by establishing a baseline level of trust.

**Table 1.** Practical Aspects and Examples of Level 1 and Level 2 Approaches

Level	Behavior	Why	Example
1. Trust	Gaze into the patient or family members' eyes	This is one of the neurobiological behaviors signaling trust, needed in any cooperative behavior. Promotes oxytocin secretion in animals, decreasing stress and increasing social bonding.	Looking deep in the patient's/relative's eyes during communication
1. Trust	Make a commitment about important core values	Reaffirming core values can improve both trust and chances of conflict resolution	“We would like to start from the very beginning, making two commitments to you. We will always tell you the truth, whether bad or good,, and we are here to help you achieve what is important to you.”
1. Trust	Check on reception	Allow space for divergent opinions or for commitment	“Does this sound ok to you?” Some patients might say, “Thank you doctor, but I already know enough. Could you talk to my daughter?” signaling their information preference. Many other patients and families express gratitude and relief. Whenever direct eye gaze decreases, we reassess trust, reconnecting with these values. Example: “I'm sorry to share this difficult news. I promised you I would always tell you the truth. And we are here for you, to work for what is most important to you, ok?”
2. Emotional resonance	Ask about concerns/worries	The goal is not just to understand the cognitive aspects of the patient/family's perception. The main goal is to tune into their emotion. Emotional empathy is the basis of caring, and a common mammalian behavior that reduces distress and provides relief to both parties involved.	“Would you be willing to share with me what has been going on with your [heart failure, cancer, etc.]? And what are you most worried about?”
2. Emotional resonance	Listen, attuning one's own emotions to the patient's/family's	In primates, those who are able to regulate their own distress are the ones able to console others. In a patient–clinician relationship, emotional self-regulation is needed for active listening. Then, emotional attunement enables empathy. Understanding which emotion is ours, which is theirs, and reminding ourselves of our goal for the encounter may help clinicians to regulate our own emotions and connect more deeply. Moreover, conflicting goals inhibit emotional resonance and empathy. Instead, we can self-regulate our own emotion by remembering that emotional attunement facilitates cooperation and conflict resolution, and hope is a natural response to fear. Thus, we do not need to “fix” or adjust hope. We can simply focus on emotional connection, which reduces fear.	“I know my mom's going to get her miracle!” Clinicians who hear this phrase can self-regulate our own emotion (often anxiety, related to the concern that “Oh, they did not understand me!)” Instead, we can recall that hope is a natural response to fear, and focus on tuning into the family's hope for an impossible solution (the definition of a miracle). Patient: “I am worried I'll die.” This may evoke a strong emotion in clinicians, and anxiety to “fix it” or change the subject. Instead, focus on regulating our own emotions, tuning into the patient's fear, and remembering that this fear is a normal emotional response and that attuning to it may attenuate it.
2. Emotional resonance	Resonate with the emotion expressed.	Connect, by repeating the emotional content in a lower tone, especially hope and fear.	“We will celebrate together if a miracle happens.” Or “This must be so hard to feel. I promise I am here with you every step of the way.” Or “I see this is important to you. It will be important for us too.” Or “I understand this worries you. Thank you for sharing with us. We will be very mindful of that.”
2. Emotional resonance	Ask—listen—resonate	Repeat the process until there is a connection and the patient/family exits the fight/flee mode. Humans experiencing dread have reduced activity in the frontal cortex (which is required for planning and reasoning). Thus, when someone is terrified, in a fight/flee mode, they are not able to weigh treatment options as they normally would.	“Could you tell me more about that?” Or “And what else worries you?” Or And what else are you hoping for?

Within the cultural context of Brazil, it can also be helpful to include direct eye contact with patients and their families to help establish trust. Within neurobiology, direct gaze from one animal to another is an integral part of facial expressions that can signal either cooperation or conflict. For example, it has been shown that the monkey amygdala contains neurons that respond selectively when a monkey makes direct eye contact with another monkey.^4^ These same interactions have also been shown to result in oxytocin secretion, leading to an attenuation of the hypothalamic-pituitary-adrenal axis, resulting in decreased stress and increased social bonding.^8,9^ Therefore, patients or family members staring down or to the side of the room can be a signal of mistrust. As clinicians seek to regain trust, a shared mutual gaze may be an early sign that trust is developing.

While Level 1 is the starting place of the hierarchy of communication needs, there may be times when mistrust and emotions are so high that violence and mistreatment explode as behaviors from patients/families toward clinicians. This is shown in the hierarchy model below level 1 (labeled: the toxic level), as one cannot meaningfully build trust and attune to emotions atop such an unstable foundation. Pushing for a serious conversation when patients/families are so activated that they are unable to engage respectfully is neither psychologically safe nor clinically effective. It may be better to pause the conversation and agree to speak further when all parties are able to engage respectfully, returning with the goal of working together to establish trust.^10^

### Level 2: Emotional resonance

After establishing trust, strong emotions such as fear and anger can still impact efforts to effectively communicate with patients/families to make shared decisions. It is well recognized that when one feels threatened, fight, flight, or freeze behaviors take over, and frontal cortex activity decreases.^1,4,11^ This results in a decreased capacity to engage in cognitive reasoning. Functional MRI studies have confirmed this phenomenon, noting increased blood flow to the amygdala and decreased blood flow to the frontal cortex in response to fearful stimuli.^12^ This can sometimes be seen when patients oscillate between expressions of hope (i.e., high emotion and low cognition) and realism (low emotion and high cognition).^13–15^ When facing difficult new information, patients will often pivot away from the challenging cognitive information they have heard as they begin to experience strong emotions, such as fear or anger. This shift may be observed as patients and their families expressing hope for a very positive and highly unlikely outcome, such as a cure for a metastatic cancer. Emotional resonance encourages clinicians first to give space for emotions to be uncovered, followed by a deliberate response to the emotion via alignment and support.^7,16^ The goal is not to try and fix or change the emotions but rather tune in and be present with the expressed emotions. Instead of forcing patients to immediately reengage with the challenging information they just heard, clinicians can provide support by just resonating and “feeling into” the emotion of the patient.

To effectively resonate with and “feel into” the emotions of a patient, clinicians must also first recognize and manage their own strong emotions. When patients mention unrealistic hope for a miracle or their worst fears about what the future might hold, this can cause emotional distress for the clinician. Clinicians will often respond by either trying to “fix” the patient's unrealistic hope by repeating prognostic information or trying to resolve the patient's fears by describing all the interventions that can still be offered. Emotional resonance only becomes possible once clinicians are able to self-regulate their own emotions sufficiently to deeply listen and respond to the emotional experience of the patient.

The impact of this sort of empathic response is also rooted in neurobiology. Researchers have observed chimpanzees, canines, elephants, and even mice provide consolation to a distressed individual. Franz de Waal explains, “[a mammalian] observer can spontaneously imitate, mimic and feel into the state of those they attend to through a neural process by which the targets' state is mapped onto the observers' perspective.”^17^ When clinicians resist the urge to adjust the expressed hope and instead align with the emotions of patients and families, this can provide the support and consolation they need to take a deep breath and face their fears that their hopes might not come true.^11,18–21^ This simple act of responding to patient's emotions has been shown to decrease anxiety and improve critical thinking and information recall.^22^ Additionally, clinicians who take the time to acknowledge emotions are also ranked as more competent by their patients and on average only adds 21 seconds to the length of a visit.^23–25^

**FIG. 2.**
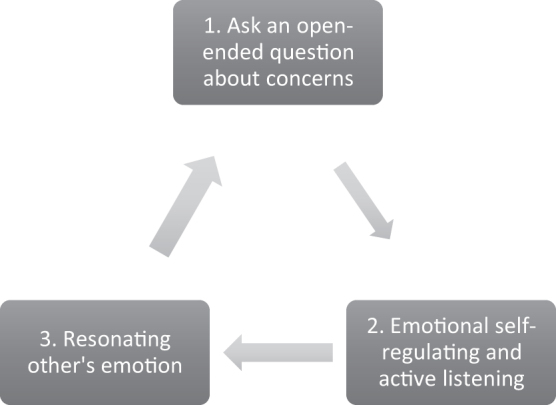
A 3-steps skill for emotional resonance.

**FIG. 3.**
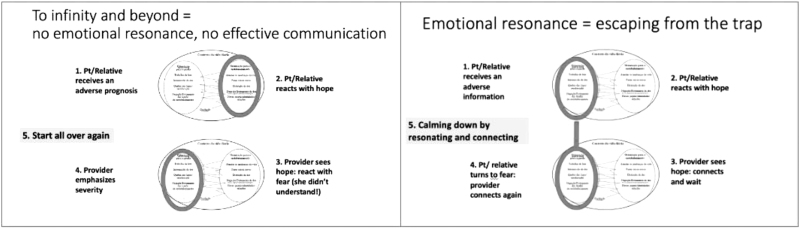
Bereavement with and without resonating emotions. Adapted from Ref.^21^ Stroebe M, Schut H. The dual process model of coping with bereavement: Rationale and description. Death Stud 1999;23:197–224.

Similar to Level 1, emotional resonance should not be a singular or sequential action on a checklist. Rather, it is a repeatable process that can be employed whenever strong emotions arise (Fig. 2). Within a typical serious illness conversation (SIC), there are often multiple key moments where difficult information is shared. When emotional reactions inevitably occur, clinicians should recognize that these are not signals that they made a mistake, but rather an indication that the patient and their family accurately heard the information. Clinicians can then promote empathy and deepen trust by resonating with these emotions (Fig. 3). Not only will this help maintain a therapeutic connection, but it also enables patients and their families to more deeply process and eventually integrate the challenging new information they are facing.

### Level 3: Synchrony/cognitive alignment

From a neurobiological perspective, it is only once trust is established and strong emotions are attuned to that patients and their families can meaningfully participate in the more cognitive activities of traditional serious-illness communication.^26–30^ However, the cognitive sharing of information and weighing of benefits and burdens is not meant to be a separate activity—but rather a third level of an integrated whole. Just as emotional resonance requires a combination of self-regulation and intent to mimic the emotional state of the patient, so too does effective cognitive exchange require an initial self-awareness of one's own perspective followed by an intent to listen to the unique lived experience of the patient.

There are many models for SICs in the literature; most include four key steps: (1) evaluating patient prognostic awareness and information preferences, (2) sharing prognostic information, (3) exploring patient goals and values in relation to the specific clinical context and prognostic information shared, and (4) making a medical recommendation for next steps in the patient's care based on their expressed goals and values. Though these same 4-steps remain essential to any SIC, the hierarchy of communication needs emphasizes that mistrust and strong emotions can recur throughout this conversation. When challenging information is shared, patients and families can react with strong emotions or even begin to lose trust. A core teaching point of the hierarchy of communication needs is to emphasize that clinicians might need to return to Level 2 or even Level 1 multiple times throughout the course of a conversation. It can even be helpful to implement one preplanned pause after the delivery of prognostic information. This is labeled as a retreat because it can feel like going backward to clinicians, though demonstrating that this retreat is expected and planned for within the model emphasizes that it is a valuable opportunity to reaffirm trust and emotional resonance. Throughout a conversation, clinicians should be prepared to pause and return to lower levels as needed, recognizing that meaningful shared decision-making is inherently dependent on patients feeling psychologically supported and in alignment with their clinicians.

## Discussion

The unique high-pressure setting of a COVID ICU at the peak of a global pandemic led to the development of a novel framework for SIC that describes a hierarchy of communication needs for patients and families. Though this framework was initially developed to respond to Brazil's unique cultural context, its structure might also suggest ways in which SIC could be adapted to better meet the needs of diverse patient populations globally.

Though cultural humility has long been recognized as a core principle of PC practice, much of the SIC literature remains heavily weighted toward a Western, low-context, and biomedical perspective.^31–33^ This perspective includes an emphasis on patient autonomy, the primacy of cognitive information, as well an assumed high level of trust between patients and clinicians. Therefore, most standard communication strategies follow a rational, stepwise approach of exchanging cognitive data, reviewing goals and values, and then outlining a recommended care plan. However, as was quickly recognized within the COVID ICU in Brazil, this logical approach can quickly break down for a variety of cultural-related reasons.

Communication discordance between clinicians and patients is often driven by cultural differences in communication and the meaning of illness.^32^ For example, many cultures may emphasize the role of miracles and the importance of maintaining faith when faced with a life-limiting illness. Additionally, what clinicians may sometimes refer to as “denial” may sometimes be a cultural resource for coping that patients and families utilize to integrate the reality of death slowly over time.^13^ Lastly, mistrust of medical institutions based on historical and ongoing inequities and injustices can also lead to discordance between clinicians and patients. For example, though many studies have suggested that African American and Latinx patients are more likely to prefer aggressive treatment at the end of life, this conclusion fails to consider how mistrust toward the medical community may impact these perspectives.^32^

A traditional, mostly cognitive approach to communication in the face of these cultural differences is inadequate to foster the culturally humble inquiry that is required. When faced with discordance in the meaning of illness, clinicians will often make multiple attempts to repeat or rephrase the same concrete, intellectual data to try and improve comprehension. This can result in worsening emotional distress for patients, pushing them toward the coping strategy of rationalization. When patients are faced with affective distress without the proper space to process their emotions, they may subconsciously change their attitudes or preferences without deliberative thought or specific intention to reduce cognitive dissonance.^14,34^ If clinicians move forward to the cognitive decisions at hand before first giving space to identify and respond to emotions, patients can become more rigid and less likely to engage in the measured, cognitive weighing of risks and benefits that is necessary to make informed decisions. Though especially common when there are significant differences between the cultural lens of the clinician and patient, this same pattern of fractured communication can occur between any two individuals.

The hierarchy of communication needs provides a clear roadmap for how clinicians can avoid this common pitfall. It incentivizes clinicians to step back from the cognitive, biomedical model and consider the broader, more complex emotional experience of patients and families. And if still facing resistance, it guides them to consider the ways in which differing views of authority and trust might be impacting the conversation. It also provides this guidance within a clear structure and with concrete instructions for how clinicians can move between levels of the hierarchy to make it more easily understood and implemented. These steps are not meant to be a comprehensive strategy for providing culturally humble care—as this requires a broader analysis of patients' attitudes, beliefs, and decision-making style in addition to their specific race, ethnicity, language, or immigration status.^32^ However, we hope this practical framework can serve as a scaffolding for more culturally humble communication.

To date, the communication strategies most represented within the PC literature have been created by clinicians from the United States or Western Europe. Though intended to be applicable to all patient populations, they have an inherent bias toward a low-context culture where meaning is explicit and cognitive knowledge is valued above all else. To better meet the needs of all patients, including those from diverse backgrounds, additional novel communication strategies that are created by and for these same populations are needed.^35^ The hierarchy of communication needs is one such approach that seeks to bridge this gap.

## Conclusion

The specific cultural context of Brazil at the peak of the COVID pandemic revealed the shortcomings of a primarily cognitive communication style rooted in the Western biomedical model. Further research will be needed to consider how this communication strategy might be applied more broadly. Though first developed in Brazil, this strategy to establish trust, attune to emotions, and then engage in cognitive exchange has now been applied in the United States as well and could likely be applied to many other settings in which more traditional SIC models may falter when a specific focus on earning trust is needed.

## Funding Information

No funding was received for this article.

## Authors Disclosure Statement

No competing financial interests exist.

## Abbreviations Used

PC: palliative care
SIC: serious illness conversation
